# Psychosocial problems among individuals with substance use disorders in drug rehabilitation centers, Nepal

**DOI:** 10.1186/s13011-016-0072-3

**Published:** 2016-08-15

**Authors:** Anju Poudel, Chandrakala Sharma, Sital Gautam, Amrit Poudel

**Affiliations:** 1 Department of Nursing, Nepal Medical College, Kathmandu, Nepal; 2 Tribhuvan University, Institute of Medicine, Nursing Campus Maharajgunj, Kathmandu, Nepal; 3 Panchamrit Research Center, Pokhara, Nepal

**Keywords:** Substance use, Substance use disorders, Psychosocial problems

## Abstract

**Background:**

Substance use is associated with numerous undesirable short and long term consequences. The individual not only suffer from physical and psychological problems but also loses the ability to interact with family, peers and society. The present study aims to identify the psychosocial problems and associated factors among individuals with substance use disorders.

**Methods:**

The study was conducted using descriptive cross sectional research design in different drug rehabilitation centers of Nepal. Probability proportional to size sampling technique was used to select the drug rehabilitation centers and individuals diagnosed with substance use disorder but free from any substance withdrawal features, admitted in rehabilitation centers within 3 months and clients above 15 years of age were included in the study (*N* = 204). A standard tool Drug Use Screening Inventory-Revised was used to assess the psychosocial problems and the scores ranged from 0 to 100. The data were analyzed using descriptive and inferential statistics. Linear regression analysis was conducted to identify the predictors of psychosocial problems.

**Results:**

The mean and standard deviation of overall psychosocial problems scores was 60.42 ± 19.44. Among different domains, more problems were found in substance use domain (75.00 ± 21.43) followed by school performance (64.60 ± 25.53), behavior pattern (64.53 ± 24.44), peer relationship (64.49 ± 24.91), social competence (61.30 ± 22.60), psychiatric disorder (56.83 ± 23.39), family system (48.28 ± 23.72) and work adjustment (45.90 ± 29.88) domains. Types of substance use, mode of substance use and age of initiation of substance use were the significant predictors of overall and most of the domains of psychosocial problems.

**Conclusion:**

Substance use poses significant impact on individual’s psychological and social wellbeing. Individuals using injecting drugs, initiating substances early in life, using substances many times in a day and using both licit and illicit substances had more psychosocial problems. Hence, treatment centers should take this into account.

## Background

The problem of substance abuse is an old phenomenon in the present day world. People have been using various kinds of psychotropic substances not only as a means of coping with various problems of life but also to derive pleasure out of it and to facilitate religious and ritualistic aims. It leads to addiction and has been associated with wide range of psychosocial problems.

World Health Organization estimates, the global burden of disease attributable to alcohol and illicit drug use is 5.4 %. The point prevalence of alcohol use disorders (in the population aged 15 years and over) is generally higher than the point prevalence of drug use disorders in the same population and is generally higher among men than among women [1]. World Drug Report 2013 also estimates that in 2011, between 167 and 315 million people aged 15–64 have used an illicit substance in the preceding year [2].

The estimated prevalence of alcohol use disorders and drug use disorders in Nepal for female is 0.48 % and 0.02 % respectively, and for male is 3.80 % and 0.15 % respectively [3]. A nationwide survey on current hard drug users in Nepal showed that there are altogether 91,534 current drug users in 2013 which is nearly double of 46,309 in 2007 [4].

Substance use is generally initiated in adolescence or early adulthood and is commonly associated with variety of problems [5–7]. These problems can be in any area of the client’s functioning: physical, psychological, family, interpersonal, social, academic, occupational, legal or spiritual. They can lead to physical and psychological dependence, coercing the person to continue taking the drug despite adverse consequences. Besides profound impairment and loss of physical health, people with alcohol and drug use disorders may suffer severely from psychosocial problems, interpersonal problems, loss of employment, difficulty in participating in education, and legal problems [1]. An extensive body of research has also demonstrated high rates of psychological impairment and reduced quality of life among the individuals with substance use disorders (SUDs) [8–10]. It has been stated that by the year 2020 mental and SUDs will surpass all physical diseases as a major cause of disability worldwide [3].

Social harms associated with psychoactive substance use include interpersonal problems that impact adversely on relationships with family members, friends, colleagues and members of society [11]. Likewise, heavy drinking can lead to a decline in the overall academic performance [12, 13]. It has been reported that about a quarter of college students experience difficulty with academics due to alcohol use, including earning low grades, doing poorly on tests and papers, missing class, and falling behind. Even students who do not abuse alcohol may suffer academically as a result of their peers’ drinking [14].

Literatures have demonstrated the relationship of age of initiation of substance use to the psychosocial problems. Individuals who engage in substance use early are more likely to develop harmful patterns of use, suffer from severe psychological problems, adjustment problems and school dropout [15–17]. Similarly, previous studies have identified age [18–20], gender [8, 18, 21–25], types of substance use [26–28], frequency of substance use [25, 26] and mode of substance use [9, 29] as the important variables associated with the psychosocial problems among the individuals with SUDs.

SUDs are typically long-lasting, persistent, challenging to treat and have tendency to relapse after remission. Psychosocial distress also could increase the probability of more substance use among patients with SUDs [30]. Therefore, it is worthwhile to identify psychosocial problems among the individuals with SUDs so that unique problems of those patients could be identified early and necessary intervention could be done to prevent the development of relapse and improve the patient's quality of life. Hence, the negative and long-term impact of a drug using lifestyle on various life domains urges for a shift of focus in such type of research. The purpose of the present study is to

- identify the psychosocial problems among the individuals with SUDs in various domains.
- assess the association of socio-demographic and substance use related characteristics with psychosocial problem domains.
- identify the predictors of psychosocial problems.

## Methods

A descriptive cross sectional research design was used to assess the psychosocial problems among the individuals with SUDs. Probability Proportional to Size (PPS) sampling technique was used to select the drug rehabilitation centers of Nepal. According to Central Bureau of Statistics Nepal, 2013 there are 22 rehabilitation centers operating in Kathmandu. At first, the list of the rehabilitation centers and number of clients residing in those centers were obtained. The number of individuals meeting the inclusion criteria in those centers ranged from 3 to 50. Eight centers were excluded as five centers did not give permission to conduct the study whereas three centers had very few clients (less than 5) who meet the inclusion criteria. One center was assigned for pretesting of the questionnaire. Based on the feasibility of the study, among those 13 centers, 6 centers (50 % of the total rehabilitation centers) were selected by using PPS sampling technique. Since respondents in some centers were few in numbers, further sampling technique at client level was not performed. All the respondents of the selected centers who met the inclusion criteria were included in the study and comprised of 204 individuals with SUDs.

All the centers had visiting psychiatrist and provide treatment for an average of 3 months. Centers were run by trained or experienced counselors and offered services for both licit and illicit substance users including alcohol users. However, only one center offered services for female substance users whereas other five centers enrolled treatment seeking male substance users. The study population was the diagnosed clients with SUDs, who were above 15 years of age and free from any substance withdrawal delirium or ongoing psychotic symptoms. Respondents admitted in treatment centers for more than 3 months were excluded from the study to reduce recall bias. The diagnosis of the SUDs was done by the psychiatrists present in those centers. In this study, the word injecting drug user refers to those respondents who uses only injecting drugs and both injecting as well as non injecting substances.

### Data collection procedure

Data were collected by using Nepali version, pre-tested self administered questionnaire. Questions regarding socio-demographic characteristics and pattern of substance use were developed after extensive literature review. Psychosocial problems of individuals with SUDs were assessed by using a standard tool Drug Use Screening Inventory-Revised (DUSI-R) [24], which was developed by Dr. Ralph Tarter in 1990. In this study psychosocial problems are the problems faced by individuals with SUDs in relation to different domains of DUSI-R. The decision to use this tool was based on its focus on the assessment of the psychosocial problems among the individuals with SUDs and established psychometric properties.

DUSI-R, a self-report questionnaire, quantifies severity of problems in 10 domains: 1) Substance Use, 2) Health Status, 3) Behavior Pattern, 4) Psychiatric Disorder, 5) School Performance, 6) Family System, 7) Work Adjustment, 8) Peer Relationships, 9) Social Competence, and, 10) Leisure/recreation. Based on the study objectives, only eight domain of DUSI-R (excluding Health Status and Leisure/Recreation domain) were included in the study. The questionnaire collects the information with reference to past 1 year. The tool is copyright so permission for the use of the instrument and inclusion of only eight domains in the study was taken from the owner.

Validity and reliability of the DUSI-R have been documented [24, 31]. For the use of the tool in Nepalese context, first forward translation (from English to Nepali) then backward translation (Nepali to English) was done. Finally, the back translated text was compared with the original text and differences between these two texts were resolved through discussion between translators for ensuring semantic equivalence. The back translated tool was also sent to the original developer of the tool for verification. To identify the accuracy, adequacy and completeness of the tool, pre testing of the translated instrument was done on fifteen respondents of one of the drug rehabilitation center. On the basis of pretesting, necessary modification were made in part I and II of the instrument. Cronbach’s alpha coefficient was computed to determine the reliability of translated (Nepali version) DUSI-R and was 0.924 which showed a high degree of internal consistency among 127 items. The alpha coefficient of different domain was as follows: Substance use = 0.79, Behavior patterns = 0.84, Psychiatric disorder = 0.84, Social competence = 0.73, School performance = 0.81, Work adjustment = 0.78, Family system = 0.76, Peer relationship = 0.81.

For each domain and overall psychosocial problem, the raw score was transformed into a scale from 0 (lower) to 100 (higher). Problems in each domain, and the overall psychosocial problem density index, were determined in standard manner using the formula provided by the tool. The school performance domain was used only by those respondents who used to go to school within the past 1 year. Similarly work adjustment domain was used by those respondents who were engaged in any form of work within the past 1 year. So, for the calculation of overall problem density index in those respondents who do not have to answer the questions regarding that domain, the denominator was adjusted.

### Statistical analysis

Data were analyzed using Statistical Package for Social Science 20 (SPSS 20). Data were interpretative as higher the score higher the psychosocial problems. As the normality test of the data revealed normal distribution of the data, parametric tests were used for the analysis. In descriptive statistics frequency, percentage, mean, range and standard deviation (SD) were used where as in inferential statistics to assess the association between different variables and psychosocial problems, t-test and one-way analysis of variance (one-way ANOVA test) was used. In all the inferential statistical procedures, *p* value of 0.05 or less (*p* ≤ 0.05) was considered statistically significant.

Furthermore, backward multiple linear regression model was constructed to identify the predictors of each psychosocial problem domain. For each psychosocial (DUSI-R) domain the initial model included all the variables that were found to be significantly related in the univariate analysis. Variables entered the model with α ≤ 0.05, and they were removed with α > 0.10. *R*^2^ was estimated for the final models to determine the amount of variance in each domain of the psychosocial problem score explained by the predictor variables.

## Results

### Characteristics of the respondents

Table 1 and 2 shows the socio-demographic and substance use related characteristics of the individuals with SUDs respectively.

**Table 1:** Socio-demographic characteristics of the respondents

*n* = 204			
Socio-demographic characteristics		Frequency	Percentage
Age in years			
< 20		38	18.6
20–29		98	48.0
30–39		38	18.6
≥ 40		30	14.7
Mean Age ± SD	29 ± 9.82 years		
Min.-Max.	15–68 years		
Sex			
Male		185	90.7
Female		19	9.3
Education			
Primary level		17	8.3
Secondary level		76	37.3
Intermediate level		77	37.7
Bachelor and above		34	16.7
Occupation			
Agriculture		7	3.4
Business		41	20.1
Service		23	11.3
Labors		13	6.4
Unemployed		56	27.5
Student		64	31.4

**Table 2:** Substance use related characteristics of the respondents

*n* = 204			
Substance use related characteristics		Frequency	Percentage
Age of initiation of substance use			
< 15 years		44	21.6
15–19		110	53.9
20–24		30	14.7
≥ 25		20	9.8
Mean age ± SD	17.6 ± 5.22 years		
Min. – Max.	9–57 years		
Types of substance use^a^			
Alcohol		153	75.0
Cannabis		115	56.3
Opiates		97	47.6
Tranquilizers		77	37.8
Stimulants (Cocaine, Amphetamines)		21	10.2
Inhalants		20	9.9
Hallucinogens		7	3.4
Licit and illicit substance user			
Licit substance (alcohol) user only		63	30.9
Any illicit substance user		50	24.5
Both licit and illicit substance user		91	44.6
Frequency of substance use			
Once or less time/day		6	2.9
2 times/day		34	16.7
3 times/day		43	21.1
More than 3 times/day		121	59.3
Mode of substance use			
Injecting		53	26
Non- Injecting		151	74

^a^Multiple Responses

### Psychosocial Problem Scores of DUSI-R

The mean and standard deviation of overall psychosocial problem scores of the respondents was 60.42 ± 19.44. Among eight domains, highest score was found in substance use domain i.e. 75.00 followed by school performance 64.60, behavior pattern 64.53, peer relationship 64.49, social competence 61.30, psychiatric disorder 56.83, family system 48.28 and work adjustment 45.90 (Table 3).

**Table 3:** Psychosocial problem scores of respondents

Psychosocial problems	Mean score	Standard deviation
Domains		
Substance use (*n* = 204)	75.00	21.43
Behavior pattern (*n* = 204)	64.53	24.44
Psychiatric disorder (*n* = 204)	56.83	23.39
Social competence (*n* = 204)	61.30	22.60
Family system (*n* = 204)	48.28	23.72
School performance (*n* = 102)	64.60	25.53
Work adjustment (*n* = 144)	45.90	29.88
Peer relationship (*n* = 204)	64.49	24.91
Overall psychosocial problem	60.42	19.44

Higher score indicates higher psychosocial problems, min.: 0, max.: 100

### Association between respondent's characteristics and DUSI-R domains

In this study, significant statistical association was observed between age and all the domain of DUSI-R except social competence and school performance domain. Regarding sex, though females had higher scores on the overall and all the domains of DUSI-R, significant association was found only with psychiatric disorder domain. Education was significantly associated with only family system domain. Regarding occupation of the respondents, economically inactive group obtained significantly higher score on overall and most of the domains except substance use and school performance domains (Table 4).

**Table 4:** Association between socio-demographic variables and DUSI-R domains

Demographic variables	Psychosocial problems (mean scores)								
	SU	BP	PD	SC	FS	SP	WA	PR	Overall
Age									
< 20 years (*n* = 31)	71.39	70.32	60.80	63.59	60.59	69.46 (*n* = 28)	63.75 (*n* = 8)	77.88	67.44
20–39 years (*n* = 140)	77.66	67.10	58.92.	62.24	47.34	62.77 (*n* = 74)	49.23 (*n* = 105)	66.73	61.90
≥ 40 years (*n* = 33)	67.07	48.18	44.24	55.19	40.69	-	30.00 (*n* = 31)	42.42	47.56
^a^ *p* value	0.022*	<.001*	0.003*	0.227	0.002*	0.239	0.001*	<.001*	<.001*
Sex									
Male (*n* = 185)	74.66	63.70	55.70	60.77	47.60	63.26 (*n* = 92)	45.39 (*n* = 128)	64.16	59.71
Female (*n* = 19)	78.24	72.63	67.89	66.54	54.88	77.00 (*n* = 10)	50.00 (*n* = 16)	67.66	67.34
^b^ *p* value	0.490	0.130	0.030*	0.291	0.203	0.106	0.563	0.561	0.104
Education									
≤ Secondary level (*n* = 93)	74.55	64.40	58.22	64.05	53.84	68.37 (*n* = 37)	43.18 (*n* = 66)	63.82	61.66
> Secondary level (n = 111)	75.37	64.63	55.67	59.00	43.62	62.46 (*n* = 65)	48.20 (*n* = 78)	65.05	59.38
^b^ *p* value	0.785	0.947	0.439	0.113	0.002*	0.263	0.317	0.726	0.407
Occupation									
Economically Active (*n* = 84)	72.14	54.58	48.33	56.29	41.92	56.33 (*n* = 15)	38.31 (*n* = 77)	53.06	52.46
Economically Inactive (*n* = 120)	77.00	71.50	62.79	64.82	52.73	66.03 (*n* = 87)	54.62 (*n* = 67)	72.50	65.99
^b^ *p* value	0.112	<.001*	<.001*	0.008*	0.001*	0.175	0.001*	<.001*	<.001*

Higher score indicates higher psychosocial problems, min.: 0, max.: 100

*SU* Substance Use, *BP* Behavior Pattern, *PD* Psychiatric Disorder, *SC* Social Competence, *FS* Family System, *SP* School Performance, *WA* Work Adjustment, *PR* Peer Relationship

^a^ANOVA test, ^b^t-test *p significant at ≤ 0.05 level of significance

Respondents who had initiated substance use before 20 years, both licit and illicit substance user and injecting drug user had significantly higher problems on overall and all the domains of DUSI-R except school performance domain. Moreover, the respondents who took substances more than or equal to 3 times/day had significantly higher score on overall problem and most of the domains except on family system domain than respondents who took substances less than 3 times per day (Table 5).

**Table 5:** Association between substance use related characteristics and DUSI-R domains

*n* = 204									
Substance use related variables	Psychosocial problems (mean scores)								
	SU	BP	PD	SC	FS	SP	WA	PR	Overall
Age of initiation of substance use									
< 20 years (*n* = 154)	78.48	70.16	61.20	65.12	51.94	65.43 (*n* = 91)	52.05 (*n* = 102)	69.75	64.93
≥ 20 years (*n* = 50)	64.26	47.20	43.40	49.57	35.00	57.72 (*n* = 11)	30.95 (*n* = 42)	48.28	46.52
^b^ *p* value	<.001*	<.001*	<.001*	<.001*	<.001*	0.347	<.001*	<.001*	<.001*
Types of substance use									
Only licit (*n* = 63)	65.29	46.66	44.44	52.15	38.09	48.33 (*n* = 6)	26.89 (*n* = 58)	42.40	46.09
Any illicit (*n* = 50)	77.86	68.00	58.20	62.42	50.28	63.24 (*n* = 37)	58.16 (*n* = 24)	75.14	64.47
Both (*n* = 91)	80.14	75.00	64.67	67.03	54.23	67.11 (*n* = 59)	58.54 (*n* = 62)	73.94	68.12
^a^ *p* value	<.001*	<.001*	<.001*	<.001*	<.001*	0.212	<.001*	<.001*	<.001*
Frequency of substance use									
<3 times/ day (*n* = 40)	58.83	52.62	48.25	52.14	43.75	53.52 (*n* = 17)	26.56 (*n* = 32)	51.25	49.04
≥3 times/ day (*n* = 164)	78.94	67.43	58.93	63.54	49.39	66.82 (*n* = 85)	51.42 (*n* = 112)	67.72	63.20
^b^ *p* value	<.001*	0.001*	0.009*	0.004*	0.178	0.050*	<.001*	<.001*	<.001*
Mode of substance use									
Injecting (*n* = 53)	88.17	78.30	69.90	70.08	56.19	70.44 (*n* = 34)	70.00 (*n* = 33)	82.34	73.22
Non-Injecting (*n* = 151)	70.37	59.70	52.25	58.23	45.50	61.69 (*n* = 68)	38.73 (*n* = 111)	58.23	55.93
^b^ *p* value	<.001*	<.001*	<.001*	0.001*	0.004*	0.103	0.001*	<.001*	<.001*

Higher score indicates higher psychosocial problems, min.: 0, max.: 100

*SU* Substance Use, *BP* Behavior Pattern, *PD* Psychiatric Disorder, *SC* Social Competence, *FS* Family System, *SP* School Performance, *WA* Work Adjustment, *PR* Peer Relationship

^a^ANOVA test, ^b^t-test *p significant at ≤ 0.05 level of significance

### Multivariate analysis of predictors of different domains of DUSI-R

Individuals using substances more frequently and injecting drug users had higher probability of problem in substance use domains whereas this probability was lower in licit substance user. Behavior problem domain was positively associated with injecting drug users whereas negatively associated with age of initiation of substance use and licit substance user. Similarly, higher problem in psychiatric disorder domain was observed with injecting mode and early initiation of substance use. Licit substance user and male had significantly lower problem in psychiatric disorder domain. Social competence domain was also positively associated with injecting mode of substance use and negatively associated with licit substance user. Individuals with higher educational level and licit substance user had greater probability of having lower problem in family system domain. It was evident that none of the variables were the significant predictors of the school performance domain. Regarding work performance and peer relationship domain, injecting substance user had higher work adjustment and peer relationship problem whereas licit substance user had lower problem related to those domain. Furthermore, early age of initiation of substance use was negatively associated with work adjustment domain. Similarly, licit type of substance use, injecting mode and age of initiation of substance use were the important predictors of overall psychosocial problem score (Table 6).

**Table 6:** Multiple linear regression analysis of the predicting variables of psychosocial problems (β standardized regression coefficients and *p* value at <0.05)

Predictors	DUSI-R Psychosocial problem domains								OVERALL
	SU	BP	PD	SC	FS	SP	WA	PR	
Sex (Reference-female)			-0.220 (0.001)						
Education					-0.260 (<0.001)				
Age of initiation of substance use		-0.177 (0.010)	-0.181 (0.011)				-0.168 (0.001)		-0.171 (0.011)
Licit substance user (Reference- both licit and illicit substance user)	-0.142 (0.42)	-0.343 (<0.001)	-0.204 (0.007)	-0.214 (0.004)	-0.260 (<0.001)		-0.344 (<0.001)	-0.364 (<0.001)	-0.324 (<0.001)
Frequency of substance use	0.242 (<0.001)								
Mode of substance use (Reference-non injecting substance user)	0.241 (0.001)	0.160 (0.015)	0.227 (0.001)	0.146 (0.048)			0.251 (0.025)	0.261 (<0.001)	0.225 (<0.001)
	*R* ^2^ = 0.21	*R* ^2^ = 0.28	*R* ^2^ = 0.23	*R* ^2^ = 0.09	*R* ^2^ = 0.17		*R* ^2^ = 0.35	*R* ^2^ = 0.42	*R* ^2^ = 0.31

*SU* Substance Use, *BP* Behavior Pattern, *PD* Psychiatric Disorder, *SC* Social Competence, *FS* Family System, *SP* School Performance, *WA* Work Adjustment, *PR* Peer Relationship

## Discussion

In this study, the mean overall psychosocial problem score of individuals with SUDs was 60.42 which was greater than the mean score (43.08) obtained in the study conducted among Turkish male adolescence [5]. Both the study highlighted higher problems in substance use and peer relationship domains while less in work adjustment domain though the score were inconsistent. The findings of this study also revealed that substance use affects not only on the individuals interpersonal relationship but also on behavioral, emotional and academic performance which was also supported by studies conducted in Brazil, Pakistan and New Zealand [6, 7, 32]. However, the other study reported that the level of school achievement of adolescents with SUDs is neither significantly lower nor the prevalence of learning disabilities higher, when compared to adolescents without SUDs [33].

Moreover, significant differences existed among the respondents of different age group in overall problem score and different domains except social competence and school performance domains. Compared to other age group, respondents below 20 years had higher score on overall and all the domains except substance use domain. This finding reveals that adolescent substance users have more psychosocial problems than adult substance abusers. Contrary to this finding, the other study revealed higher psychosocial problems in young adult followed by mid adult and lesser in 12–18 years youth [18]. Similarly, study conducted in Nepal reported that, among the patients with SUDs, majority had depression and were adult and older group than adolescence [19]. This is probably because; these two studies were conducted in community and hospital setting respectively whereas the current study was done in rehabilitation centers.

Regarding sex, females had higher score on the overall psychosocial problem score and all the domains of DUSI-R. However, significant association was found only on psychiatric disorder domain. The result of this variable might have been influenced by few numbers of female respondents in this study. Various studies examining overall rates of psychiatric co-morbidity found that women substance users have more psychological problems than men [8, 21]. The findings were also supported by the study done among individuals admitted for substance use treatment in Manitoba, Canada which showed that female had significantly severe disturbance on overall and all the domain of DUSI-R except work adjustment domain [25]. Contrary to this study finding, the other study revealed higher problems among substance use disorder male than female in overall and almost all domain of DUSI-R, however significant difference were observed only on overall problem, substance use, work adjustment and peer relationship domain [24].

In this study education was significantly associated with only family system domain. Regarding occupation, economically inactive respondents obtained significantly higher score in the overall and all the domains except substance use and school performance domain. Here, work capacity, financial independence and less free time might be the possible factors contributing to the lower psychosocial problem among economically active group. Similarly, as unemployed have to depend on others for money to buy substances, they try to engage in some work but fail to continue it so that they might have more psychiatric, behavioral, peer relationship, family system and work adjustment problems. Similar finding was reported in the study conducted in Nepal, where the majority of the unemployed substance users had depression than employed substance users [19].

Regarding age of initiation, the respondents who had initiated substance use before 20 years had significantly higher problems on overall and all the domains except school performance domain than those respondents who initiated substance use at 20 years or after that. Though not significant on school performance domain the problem was higher in same group. This finding is supported by the study conducted in Virginia which showed that early drinking is strongly related with severity of substance use problems and reduced engagement in social activities including work and school problems [17]. The other studies conducted in New Zealand and UK also supported the findings [12, 13, 34, 35].

The variable like types of substance use had significant influence on overall problem and different domains except school performance domain. The study findings revealed that respondents who use both licit and illicit substances had higher score on overall and almost all domains whereas licit substance users (only alcohol users) had lower score on overall problem as well as all the domains than other two groups. The study conducted in Norway also supported the current study findings which revealed that psychosocial symptoms load was higher in the groups with high consumption level of illicit substances compared to those who mainly had high consumption levels of alcohol [27]. However, contradictory to the above findings, study done in UK did not find any significant differences between alcohol dependent clients and drug and alcohol dependent clients on the measure of quality of life at different domains [8].

The frequency of the substance use was found to be significantly associated with the overall problem and different domains expect family system domain. Respondents who took substances more than or equal to 3 times/day obtained higher score on overall problem and all the domains. This findings has been supported by other study which showed that individuals who reported using cannabis more than 10 times in the past year were at greater risk of substance use problem and were more likely to drop out of school and be unemployed than those who used less than 10 times [36].

Similarly, compared to non-injecting substance users, injecting substance users had significantly higher score on overall as well as all the domain of DUSI-R except for school performance domain. The study conducted in Nepal also supported the findings which revealed that all the patients using intravenous drug had depression whereas only 66 % of the non injecting substance users had depression [19]. Likewise, the other study conducted among hidden homeless injecting drug users in a Canadian community also showed that compared to non injecting substance users, injecting drug users had more family and social relationship problems, less social support and more behavioral problems [29].

Though many variables were identified as the associated factors of psychosocial problem domains, further linear regression analysis revealed mode and type of substance use as the important predictors of overall and most of the domains (substance use, behavior pattern, psychiatric disorder, social competence, work adjustment and peer relationship) of DUSI-R. Injecting drug users had higher problems compared to non-injecting substance users and licit substance user had significantly lower problems compared to both (licit and illicit) type of substance user. The present study also suggested frequency of substance use as positive predictor of SUD domain and age of initiation of substance use as the negatively associated predictor of behavior pattern, psychiatric disorder, work adjustment domains and overall psychosocial problem score. Male and individuals with higher educational level were identified as the significant predictor of lower problem in psychiatric disorder and family system domain respectively. None of the variables were the significant predictors of school performance domain. It might be due to lack of statistical power as a result of small sample size. The school performance domain was used only by those respondents who used to go to school within the past 1 year.

The finding of this study has to be considered with reference to its limitations. The female substance users were very few in number and were from only one center. Thus caution should be used in generalizing the findings related to sex variable. Due to few numbers of respondents in some centers, stratified sampling technique was not applied and analysis was also performed by combining the respondents from different centers though there were some differences among the centers. It might have reduced the strength of the study. The other limitation is that the power analysis was not carried out; thus statistical power of the study may be open to question. Regarding frequency of substance use, the present study is based on number of times of substance use per day without much regard to quantity of substance use. This could have influenced the information regarding that variable. There is possibility of recall bias as data was collected within the reference period of 1 year.

## Conclusion

Individuals with SUDs have more psychosocial problems and the problems are in different areas of life, so they need support and counseling for those areas. Injecting drug users, respondents initiating substance use early in life, using substances many times in a day and using both licit and illicit substances, are at greater risk of having higher psychosocial problems. Since substance use is linked to diverse areas of life and has been associated with various factors, treatment should be multifaceted in comparison to routine care only and should take into account of differences in socio-demographic and substance use related characteristics.

## Abbreviations

ANOVA, analysis of variance; BP, behavior pattern; DUSI-R, drug use screening inventory-revised; FS, family system; PD, psychiatric disorder; PPS, probability proportional to size; PR, peer relationship; SC, social competence; SD, standard deviation; SP, school performance; SPSS, statistical package for social sciences; SU, substance use; SUDs, substance use disorders; WA, work adjustment
